# Supplementary material for: Acrylamide Exposure from Common Culinary Preparations in Spain, in Household, Catering and Industrial Settings
Source: Foods. 2021 Aug 26;10(9):2008. doi: 10.3390/foods10092008 (PMC8467121; doi:10.3390/foods10092008)
Supplement: Supplementary file 1 [file foods-10-02008-s001.zip › foods-1309947-supplementary.pdf]

Acrylamide Exposure from Common Culinary Preparations in Spain, in Household, Catering and Industrial Settings

Lucía González-Mulero, Marta Mesías *, Francisco J Morales, Cristina Delgado-Andrade

SUPLEMMENTARY

Table S1. Ingredients and culinary treatments used in the preparation of different food groups analyzed in the present study.

Food group	Ingredients	Culinary treatment*
Potato-based food		
Chips	Potatoes, salt	Frying
Spanish omelette	Potatoes, salt, eggs and optionally (onion)	Frying
Cereal-based food		
Torrijas	Sliced wheat-flour bread, milk, eggs, sugar, cinnamon	Frying
Sponge cake	Wheat flour, sugar, eggs, baking powder, and optionally (shortenings, vegetable oils)	Roasting
Food based on cereal mix with meat, fish or vegetables		
Breaded fillet	Chicken fillet, breadcrumbs, wheat flour, eggs	Roasting/frying
Ham and cheese fillet	Cooked ham, cheese, pork fillet, breadcrumbs, wheat flour, eggs	Frying
Pizza	Pizza dough, tomato, cheese and other ingredients such as oregano, meat, cooked ham, bacon, olives, tuna or mushrooms	Roasting
Puff pastry pies	Puff pastry, tomato, tuna, red pepper	Roasting
Patties	Dough of patties, tomato, tuna, hard-boiled egg, red pepper	Roasting/Frying
Migas	Wheat-flour bread, green pepper, bacon, chorizo sausage, garlic, paprika, and optionally (grapes, pork meat)	Sautéed
Croquettes	Bechamel sauce (milk, wheat flour), breadcrumbs, egg and other ingredients such as cooked ham, bacon, chicken, tuna or cheese	Frying

*All the frying treatments used vegetable oils.
